# Mycobacterium tuberculosis and non-tubercular mycobacterium infection in women with unexplained infertility from eastern India

**Published:** 2018-09

**Authors:** Tridip Chatterjee, Ashim Kumar Basak

**Affiliations:** 1 *Suraksha Genomics (R & D Division of Suraksha Diagnostic), Salt Lake, Kolkata, India* *.*; 2 *Department of Molecular Biology, Institute of Genetic Engineering, 30, Thakurhat Road, Kolkata, India.*

**Keywords:** GTB, NTM, Real-time polymerase chain reaction, Unexplained infertility

## Abstract

**Background:**

Genital tuberculosis (GTB) is an important cause of female infertility, especially in developing countries. The positive results of polymerase chain reaction (PCR) in endometrial GTB in the absence of tubal damage raise the possibility of the detection of sub-clinical or latent disease, with doubtful benefits of treatment.

**Objective:**

This study aims to evaluate the Mycobacterium tuberculosis (MTB) and Non-tubercular Mycobacterium (NTM) infection by using Real-PCR technique in the menstrual blood samples of 120 unexplained infertile women.

**Materials and Methods:**

In this cross-sectional study, 120 infertile women with unexplained infertility aged 20-35 yr old and normal hysterosalpingography findings were taken. Menstrual blood in the first 12 hr of menstruation containing the endometrial tissues from each participant was tested for MTB and NTM by Real-Time PCR.

**Results:**

Among the selected 120 patients, only two were found to be positive for MTB infection. All remaining participants were negative for MTB infection. All participants were negative for NTM infection at the endometrium.

**Conclusion:**

Although, studies have indicated that PCR is a useful method in diagnosing early GTB disease in infertile women with no demonstrable evidence of tubal or endometrial involvement, our study showed that GTB is not the major problem in women with unexplained infertility.

## Introduction

Female infertility is the outcome of a number of factors that include: problems in ovulation, tubal blockage, uterine problem, endomeriosis, pelvic disorders, endocrine disorder as well as ‘unexplained fertility’ for which medical examinations can not reveal any specific cause (1). Unexplained infertility is a medical condition in which the couple fails to conceive in spite of all standard tests being reported normal (2). Genital tuberculosis (GTB) is a major pelvic factor causing infertility that often exists without any clinical symptom and is considered as an important etiological factor in patients with unexplained infertility (3-5). GTB is mainly secondary infection acquired by hematogenous spread from extragenital sources like pulmonary or abdominal tuberculosis (3). 

Mycobacterium tuberculosis (MTB), the causative agent of genital tuberculosis involves mainly fallopian tube, endometrium and to lesser extents in ovary, cervix (6). Since it very difficult to take tissue from fallopian tube, specimen from endometrium can be taken to detect GTB (7). Compared to endometrial samples obtained by interventional procedures like curettage that can spread the existing pathology, testing of menstrual blood was proposed as a potential less invasive sample which could be easily obtained in asymptomatic cases presenting with infertility. Menstrual blood samples of infertile women are widely used for molecular diagnostic tests to diagnose endometrial tuberculosis and women are managed depending on results obtained (8). The high detection of MTB in menstrual blood can be obtained if the menstrual blood samples were taken during initial 12 hr of menstruation in which the whole endometrium was sloughed out, yielding good endometrial tissue in menstrual blood (9). It has been reported that no major difference existed in the sensitivity of detection of MTB either by using menstrual blood or endometrial sample and both were effective for the diagnosis of tuberculous endometritis (10). Although pulmonary tuberculosis can be successfully diagnosed with microscopy in acid- fast bacilli smear and Lowenstein Jesen medium, they are less applicable in GTB owing to very low MTB load in genital tissues (4). 

Rapid nucleic acid amplification techniques, such as PCR has many advantages, such as only 10 CFU/ml is detectable and very small time is needed for obtaining results. Advanced PCR techniques like Real-time PCR (RT-PCR) markedly reduce the incidence of false positive results as amplification and detection takes place in the sample reaction tube (11). Furthermore, turnout times i.e. total amount of time from the beginning of analysis to the issuance of the final report, for RT-PCR are substantially lower (only a few hours) than the conventional testing methods (many days) making this technique cost saving and enable the physicians to start appropriately directed therapy much sooner (12). It has been reported that a number of Non-tubercular Mycobacterium (NTM) existed in the endometrial tissues of females with infertility. Thus diagnosis and differentiation of NTM are also very important to afford appropriate therapy for the patients (13).

In this present study an initiative has been taken to detect MTB and NTM by RT-PCR in the early menstrual blood and endometrial contents within it of 120 unexplained infertile females to find association of GTB with their infertility. The detection employed nested PCR for the detection of MTB and NTM. The first amplification was subjected for sequence-specific amplification of MTB and NTM and the second amplification was a nested PCR reaction in order to achieve the maximum sensitivity and specificity of the test.

## Materials and methods

This cross-sectional study included the infertile women aged between 20-35 yr, who came to us for diagnosis of their GTB infection (total no. 120). None of the selected women had any history of endocrine disorders, polycystic ovarian syndrome or any known cause of infertility. Any women with a prior history of tuberculosis, contact history of tuberculosis and chest radiological findings were excluded from this study. 

**Specimen collection**

Specimens were collected within first 12 hr of the onset of menstruation. The patients were given a sterile container, where they themselves collected the menstrual secretions, which contain a heterogeneous mixture of endometrial tissues and menstrual blood. The patients were advised not to have any physical intercourse before 48 hr of sample collection and also advised not to use any gel or cream at vaginal area prior collection of sample.

**DNA extraction**

The Mycobacterial DNA extraction was done using commercially available kit from 3B Black Biotech Ltd. in the following steps:

- Sample lysis
- Sample neutralization
- Adjusting DNA binding conditions
- Binding of DNA
- Washing silica membrane
- Eluting highly pure DNA

The samples were centrifuged and endometrial tissues, present in menstrual secretion were finely chopped with sterile surgical blade. Following this 100 μl lysis buffer was added to the pellet and the sample was incubated at a boiling water bath (100^o^C) for 15 min. After this step 100 μl neutralization buffer was added to the samples, mixed well and centrifuged for 2 min at 10,000 rpm. After centrifugation 200 μl of supernatant was transferred into a new 1.5 ml microcentrifuge tube and 250 μl of buffer was added to this for facilitating the DNA binding condition. After this step 250 μl of ethanol (96-100%) was added to samples and the mixture was vortexed vigorously (for 10-20 sec). 

Finally, the mixture was pipetted out into the spin columns (silica gel based) placed into a 2 ml collection tube. Then the spin column was centrifuged for 1 min at 10,000 rpm and the flow-through was discarded. Now the DNA has bound to the membrane. Now it needs to be washed couple of times with wash buffers and finally DNA was eluted with 50 μl elution buffer after incubating the membrane for 1 min at 70^o^C. After elution the DNA were stored in -20^o^C till further use. The membrane was washed couple of times with wash buffers and finally DNA was eluted with 50 μl elution buffer after incubating the membrane for 1 min at 70^o^C. After elution the DNA were stored in -20^o^C till further use.

**RT- PCR**

The RT-PCR was done using the commercially available kit from 3B Black Biotech Ltd. This RT-PCR kit is approved by CE and IVD. The PCR Mix was prepared as per Table I in 0.2 ml PCR tubes. To 21.5 μl of PCR reaction mix, 8.5 μl of extracted DNA samples were added and the final volume was 30 μl. In each batch of PCR run one POSITIVE and one NEGATIVE control was done. For, POSITIVE control, 1 μl of positive control (provided by the kit manufacturer) was added and the final volume was made up to 30 μl with nuclease-free water. For, the NEGATIVE control, 8.5 μl of negative control was added. 

**RT-PCR machine program set up**

We have used Rotor Gene Q (6 plex) RT-PCR machine and the machine was programmed as per Table II.

**Channel selection**

The settings for channel selection were defined in the PCR machine as per Table III.

**Ethical consideration**

The whole study protocol was approved by the Institutional Ethics committee. Oral Consent was taken from each of the participants for their clinical data can be used for research purpose.

**Table I:** PCR mix recipe

**Serial No.**	**Reagents**	**Amount** **(for 1 reaction)**
1	Multiplex master mix	14 μl
2	MTB primer probe mix	3 μl
3	NTM primer probe mix	3 μl
4	Internal control primer probe mix	1.5 μl

MTB: Mycobacterium tuberculosis

NTM: Non-tubercular mycobacterium

**Table II:** Nested PCR cycles and Dye acquisition condition

**Steps**	**Temperature (°С)**	**Time**	**Dye acquisition**	**Cycles**
1	94	10 min	-	1
2	94	30 sec	-	10
	68	30 sec	-	
	72	45 sec	-	
3	94	30sec	-	32
	59	60 sec	YES (HEX, FAM, TEX RED)	

Tex Red: Orange florescent dye

HEX: Yellow florescent dye

FAM: Green florescent dye

**Table III:** Channel selection settings

**Detection**	**Detector name**	**Reporter dye**	**Dye colour**
MTB complex specific DNA	MTC	HEX/VIC	YELLOW
*Mycobacterium* genus specific DNA	*Mycobacterium* genus (MTC+NTM)	FAM	GREEN
Internal control	IC	Tex Red	ORANGE

MTB: Mycobacterium tuberculosis

MTC: Mycobacterium tuberculosis complex

NTM: Non-tubercular mycobacterium

HEX/ VIC: Yellow florescent dye

FAM: Green florescent dye

Tex Red: Orange florescent dye

IC: Internal control

## Results

**Figure 1:**
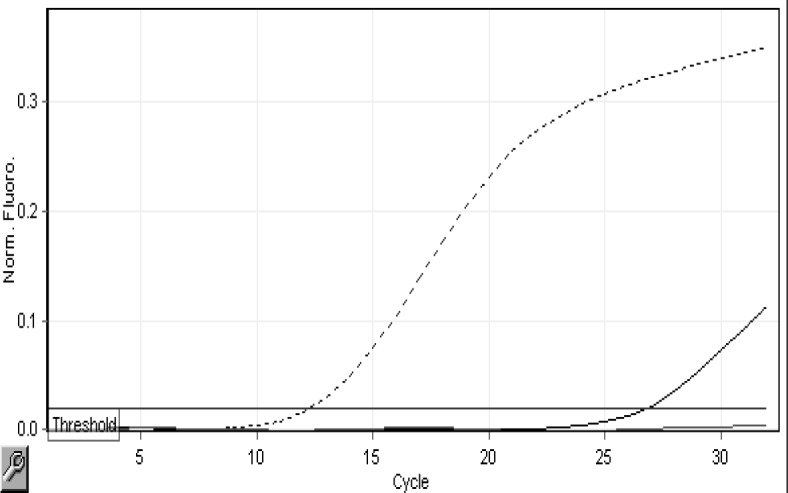
Result showing the amplification of one POSITIVE sample for MTB in Yellow (HEX) channel

Note: [Positive control (PC) is amplified (Ct 12.33) and Negative control (NC) is not amplified, which assertain the accuracy of the PCR run. The sample get amplified with Ct 26.82, at 0.02 threshold].

**Figure 2:**
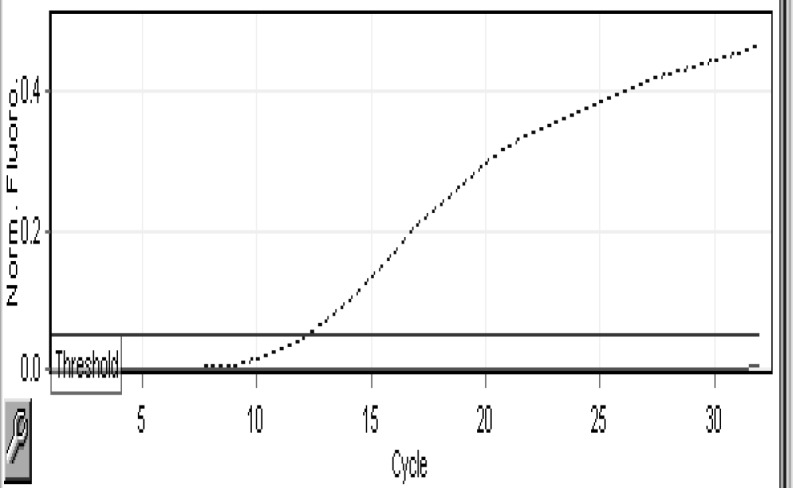
Result showing the amplification the same sample as NEGATIVE for NTM in Green (FAM) channel.

Note: [Positive control (PC) is amplified (Ct 12.08) and Negative control (NC) is not amplified, which assertain the accuracy of the PCR run. The same sample was not amplified, which ensures absence of NTM].

We were very strictly followed the results and data of the RT-PCR runs. If there was proper amplification of Internal Control (IC) in the TEXAS RED channel, then only it was taken for further analysis for detecting the presence of MTB or NTM. Out of our analyzed 120 cases, we have got M. tuberculosis in 2 samples. 118 were free from any kind of MTB or NTM infection. The Figure 1 and 2 shows the amplification of one positive sample for MTB in Yellow (HEX) channel and Negativity of the same for sample for NTM, which is evident by the graph of Green (FAM) channel.

## Discussion

The incidence of female infertility is rising throughout the world of which 30% of the infertile couple diagnosed with unexplained infertility (14, 15). Although female GTB is considered as the major cause of unexplained infertility (16), its prevalence is rare in the western world but is common in developing countries (17). Young women with age ranging between 20-40 yr are most susceptible to GTB (3). The prevalence data of GTB among infertile women vary considerably in developing countries such as 3-26% in India, 2.4-20% in Pakistan, 6.9% in Yemen etc. (18). Diagnosis of MTB by PCR is more sensitive than other conventional techniques and molecular diagnosis of genital tuberculosis by an improved PCR technique i.e. RT-PCR offer an extremely sensitive and specific technology that require miniscule amount of genetic material of the organism under investigation to determine its existence. 

In this present study, we have analyzed first 12 hr menstrual blood which is reported to be a good source of endometrial tissue (9) from 120 females with unexplained infertility, having ages between 20-35 yr to determine the presence of MTB and NTM simultaneously. Out of our analyzed 120 cases, only 2 samples were positive for MTB and none of the samples exhibited any association with NTM infection. Although our result i.e. 1.6% positivity for MTB, does not match the general trend of the association of genital tuberculosis infection in females with unexplained infertility in developing country like India, similar report is also available from other developing countries, where no or negligible MTB positive cases were found to be the cause of unexplained infertility. 

For example, in a cross-sectional study in Iran endometrial biopsy samples of 144 infertile women with unexplained infertility aged 20-35 yr, the PCR results of endometrial specimens were negative in all cases indicating that there was no GTB infection in these patients (1). Therefore, it is reasonable to consider that GTB could not be taken always as a major factor in contributing unexplained infertility in women. Furthermore, our result also could not establish any association of NTM infection with infertility among the patients.

## Conclusion

Although MTB is quite prevalent in developing countries like India, but GTB is not that much common as compared to pulmonary tuberculosis (19). Anyway, it also cannot be ruled out from the causes of unexplained infertility, as we got evidence of presence of MTB in 1.6% cases. Thus it can be concluded that in the absence of GTB, clinicians should carefully monitor the other causes mentioned just now for diagnosing the etiology of unexplained female infertility to provide proper therapeutic measures to the patients.
